# Intrathoracic extrapulmonary hydatid cysts

**Published:** 2012-09-08

**Authors:** Fouad Atoini, Aziz Ouarssani, Moulay Ahmed Hachimi, Fatima Aitlhou, Mustapha Idrissi Rguibi, Abdelaziz Hommadi

**Affiliations:** 1 Department of Thoracic surgery, Military Hospital Moulay Ismail. Postal box: S 15. Meknes. Morocco; 2 Department of Pulmonology, Military Hospital Moulay Ismail. Postal box: S 15. Meknes. Morocco; 3 Department of Anaesthesiology, Military Hospital Moulay Ismail. Postal box: S 15. Meknes. Morocco; 4 Department of Radiology Military Hospital Moulay Ismail. Postal box: S 15. Meknes. Morocco

**Keywords:** Hydatid cyst, thoracic hydatid cyst, extrapulmonary, intrathoracic

## Abstract

Hydatid disease caused by echinococcus granulosus is still a serious problem in both underdeveloped and developing countries. Clinical signs of the disease are not specific. Most patients have a few symptoms when a hydatid cyst is discovered. Symptoms depend on its location, size and complications. Parasite can settle in every organ and tissue in the human body. We report two cases with intrathoracic extrapulmonary hydatid cyst with multiple cysts. Pathophysiology of the mode of dissemination, and surgery are discussed.

## Introduction

Hydatid cyst disease, or echinococcus, is a parasitic disease caused by the larval stage of *Echinococcus granulosis*. It remains epidemic in Morocco and other countries. Echinococcal cysts may develop in almost any part of the body. The liver and the lung are the most commonly affected areas in adults. It is not difficult to diagnose typical pulmonary cysts. Conversely, when cysts appear intrathoracically but in extrapulmonary locations, crucial diagnostic difficulties may occur, with atypical clinical and radiological signs. Usually the diagnosis is surgical. The aim is to discuss the mode of dissemination of the parasite in these locations, and the optimal therapy for this entity.

## Patients and observations

### Case 1

**Figure 1:**
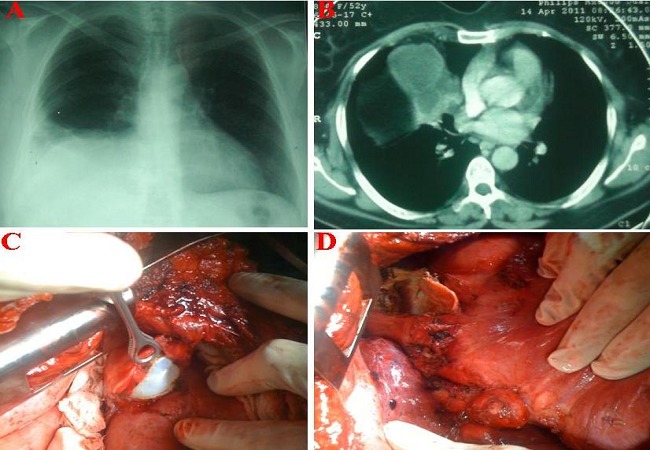
A-Chest X-ray showed large opacity of the inferior right hemithorax with elevation of diaphragm, B-Chest-CT showed cystic lesion in the diaphragm area, C-Peroperative view confirming the diaphragmatic location of the hydatid cyst, D-Peroperative view showed multiple hydatid cyst of the diaphragm

A 16-year-old female was admitted in our department for chest pain and dyspnea. Interrogation showed no medical history and the patient lives in rural area. Clinical examination was normal in particular in the chest. Chest radiograph showed two water opacity in the right thorax suggesting hydatidosis, hydatid serology was positive and chest CT evoked triple location of right pulmonary hydatidosis (Figure 1A, B). Through the sixth posterolateral thoracotomy, exploration showed location of three extrapulmonary hydatid cysts: one was located in the anterior chest wall parallel to sternal line, the second in the lateral chest wall and the third have diaphragmatic location with mediastinum and chest wall extension, this cyst compressed the lung with dense adhesions (Figure 1C, D). Subtotal cystotomy was performed for the diaphragmatic cyst because of dense adhesion to the mediastinal structures, and the two chest wall cysts were resected completely with associated pleurectomy because of pleura involvement. The whole lobes of lung were free after careful palpation.

### Case 2

**Figure 2:**
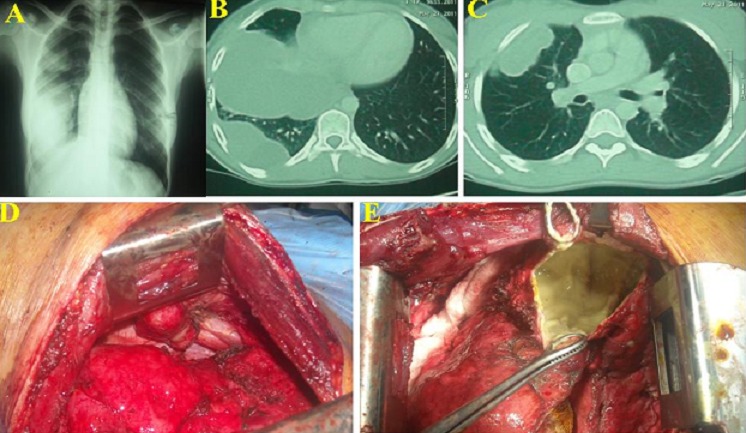
A-Chest X-ray showed multiple water opacity of the right hemithorax, B and C- Chest-CT in favour of triple location of pulmonary hydatid cyst, D- Peroperative view showed apical parietal hydatid cyst, E- Peroperative view showed large extrapulmonary hydatid cyst located between the mediastinum and diaphragm

The patient was operated 06 times for recurrent liver hydatidosis followed by albendazol therapy at 10 mg/kg/day, on the basis of chest radiograph and chest CT (Figure 2A, B, C), the most likely diagnosis advocated was diaphragmatic eventration or hernia with possible recurrence of the liver cyst, the aim of thoracotomy was to treat the diaphragmatic paralysis and resection of the cyst through this approach. Through the seventh posterolateral thoracotomy, exploration showed diffuse diaphragmatic hydatidosis associated with large eventration (Figure 2D, E). Total cystotomy of the cyst, without the cut of the diaphragm. Plication of diaphragm was performed to repair eventration. There were no diaphragmatic hernia after carefull examination and the lung was free of cyst. Albendazole therapy were administered for the two patients at 12 mg/kg/day, witch was administered for 04 cycles including free 15 day between 28 day for each cycle of treatment, tolerability of treatment was based on clinical signs and with laboratory tests including (blood count and hepatic tests).

## Discussion

Intrathoracic extrapulmonary hydatid cysts (IEHC) are very rare (5-7%) [1–6], and are found in the chest wall, mediastinal, pericardial, myocardial, pleural, lobar fissure and diaphragmatic. Dakak M et al. [2] reported the most common location in the mediastinum (42%), 27% were in the chest wall, and 24% in the diaphragm, only 6% were located in the pleural space. Unlike pleural hydatid cyst were reported to be the most common forms of IEHC (72,7%) [1, 5]. Three pathways had been explained for the development of intrathoracic hydatid cysts. In the first, embryos attach and penetrate to the mucosa of the duodenum and jejunum, enter the mesenteric venules, and proceed to the portal vein. In the portal circulation, some embryos whose diameter do not exceed 0,3 mm may pass through the sinus capillaries of the liver and, by way of the hepatic veins and vena cava, proceed to the right side of the heart and the pulmonary capillaries [7]. In the second pathway, embryos enter the lymphatics of the small intestine, proceed to the thoracic duct, to the internal jugular vein, to the right side of the heart, and to the lung [7]. The third possible route is a venal-venous anastomosis in the liver and the space of Retzius. Some researchers have supported the possibility of direct pulmonary exposure through the inhalation of air contamined with echinococcus [7]. However we found, in case 1: giant hydatid cyst develpped in diaphragm and two extrapleural hydatid cysts between the parietal pleura and endothoracic fascia, it was a primary hydatid cysts, in case 2 there are seen multiple diaphragmatic hydatid cysts, in this case the hydatidosis was secondary by dissemination because of the liver hydatid cyst, we suggest there is a different route of dissemination. As Istimangil reported [8], a dissemination pathway may be lymphatics from the dome of the liver. The scoleces had probably been transported by diaphragmatic lymphatics because drainage of lymphatics from the dome of liver and diaphragm was known to ascend anteriorly over the parasternal lymph nodes posteriorly over the intercostals lymph nodes [9]. This mechanism explains how the cysts are located parallel to parasternal lymph nodes and intercostals lymph nodes in case 1. Hydatid disease is initially diagnosed by its familiar radiographic finding in endemic areas like our country (Morocco). Currently the chest radiograph and thoracic CT are sufficient to reach a diagnosis of hydatidosis. Although serologic tests are helpful, but they have lesser value in diagnosis, because of false-negative and false-positive results [1–6]. However, in cases with unusual localisation, like reported in this paper, with atypical radiological appearance, exact diagnosis are not always reliable, and difficulties may lead to an incorrectly conceived initial surgical approach. Precise diagnosis usually occurs during surgical intervention [4], and surgical procedures are planned peroperatively.

Surgical procedures for extrapulmonary intrathoracic hydatid cysts differ from those used for pulmonary cysts. Procedures that conserve lung tissue are appropriate for most patients with pulmonary hydatid cyst. Radical procedures should avoid and surgery should preserve lung parenchyma to the maximum, cystotomy with capitonnage is the standard treatment, very rarely parenchyma is destroyed by the cyst requiring major resection [3]. However, for the IEHC forms and in the presence of surrounding tissue involvement by the cyst (chest wall, pleura, diaphragm, mediastinum) additional surgical resections are required [1–6, 9, 10]. In the chest wall locations, surgery may be decaying and associated resections consist of pleurectomy, curttage and debridment of the rib with partial or total resection, excision of soft tissue is necessary in major invasion [2, 5, 6, 9]. The diaphragmatic locations must be treated by total excision of the cyst with cut of the diaphragm and repair with suture or prosthetic mesh [2, 4–6], in case 2 the resection of the cyst was realized without diaphragmatic cut because of history of multiple surgery of the hepatic dome for liver hydatidosis and the major risk of this procedure. In the mediastinum, surgical resection should be employed without extensive excision when progression of dissection is impossible or dangerous because of dense adhesions of vital structures [11]. Because of high risk of reccurence and according to previous study and the recommendations of WHO, albendazole treatment should be applied to the patients at a dose of 10 to 15 mg/kg per day for 4 to 6 cycles, four week for each cycle with 2 weeks intervals [2, 9].

## Conclusion

Intrathoracic extrapulmonary hydatid cyst are very rare, our discussion through these two cases will give an especially insight into hydatid cyst pathophysiology, surgical particularity and difficulties in comparison of pulmonary forms.

## References

[1] Oguzkaya F, Akçali Y, Kahraman C, Emirogullari N, Bilgin M, Sahin A (1997). Unusually located hydatid cysts: intrathoracic but extrapulmonary. Ann Thorac Surg..

[2] Dakak M, Yucel O, Kavakli K, Caylak H, Gozubuyuk A, Sapmaz E, Cubuk S, Gurkok S, Sahin MA, Genç O (2009). Intrathoracic Extrapulmonary Hydatid Cysts: Review of 33 Cases. Trakya Univ Tip Falk Derg..

[3] Isitmangil T, Sebit S, Tunc H, Gorur R, Erdik O, Kunter E, Toker A, Balkanli K, Ozturk OY (2002). Clinical experience of surgical therapy in 207 patients with thoracic hydatidosis over a 12-year-period. Swiss Med Wkly..

[4] Gursoy S, Ucvet A, Tozum H, Erbaycu AE, Kul C, Basok O (2009). Primary intrathoracic extrapulmonary hydatid cysts: analysis of 14 patients with a rare clinical entity. Tex Heart Inst J..

[5] Sebit S, Tunc H, Gorur R, Isitmangil T, Yildizhan A, Us MH, Pocan S, Balkanli K, Ozturk OY (2005). The evaluation of 13 patients with intrathoracic extrapulmonary hydatidosis. J Int Med Res..

[6] Gozubuyuk A, Savasoz B, Gurkok S, Yucel O, Caylak H, Kavakli K, Dakak M, Genc O (2007). Unusually located thoracic hydatid cysts. Ann Saudi Med..

[7] Aletras H, Symbas P N, Shields T. W, LoCicero J (2000). Hydatid disase of the lung.

[8] Isitmangil T, Toker A, Sebit S, Erdik O, Tunk H, Gorur R (2003). A novel terminology and dissemination theory for a subgroup of intrathoracic extrapulmonary hydatid cysts. Med Hypotheses..

[9] Kiss F, Szent_agothai J (1964). The lymphatic system.

[10] Zendah I, Ben Saad S, Daghfous H, Ayadi A, Toujani S, Merai S, Ben M'rad S, Tritar F (2009). Hydatid cyst of the chest wall mimicking metastatic colon cancer. Rev Pneumol Clin..

[11] Traibi A, Atoini F, Zidane A, Arsalane A, Kabiriel H (2010). Mediastinal hydatid cyst. J Chin Med Assoc..

